# Electrodeposited Bi_2_Te_3_-carbon black nanocomposites with enhanced thermoelectric and mechanical properties

**DOI:** 10.1016/j.isci.2026.116977

**Published:** 2026-08-22

**Authors:** Khairul Fadzli Samat, Muhammad Afiff Alias, Muhammad Aliff Ikhwan Che Azman, Mohd Hafiz Jali, Nguyen Van Toan, Takahito Ono

**Affiliations:** 1 Fakulti Teknologi dan Kejuruteraan Industri dan Pembuatan, Universiti Teknikal Malaysia Melaka, Melaka 76100, Malaysia; 2 Fakulti Teknologi dan Kejuruteraan Elektrik, Universiti Teknikal Malaysia Melaka, Melaka 76100, Malaysia; 3 Department of Mechanical Systems Engineering, Tohoku University, Aoba-ku, Sendai 980-8579, Japan; 4 Micro System Integration Center, Tohoku University, Aoba-ku, Sendai 980-8579, Japan

**Keywords:** thermoelectric film, bismuth telluride, nanocomposite, electrochemical deposition, carbon-based nanomaterials, thermal conductivity

## Abstract

Thermoelectric thin films based on bismuth telluride (Bi_2_Te_3_) are widely used for room temperature energy harvesting, but improving electrical, thermal, and mechanical properties simultaneously remains challenging. In this study, Bi_2_Te_3_-carbon black nanoparticle nanocomposite films were synthesized by electrodeposition and then systematically characterized. Porous carbon black nanoparticle incorporation increased electrical conductivity to 1,418 S cm^−1^, more than double that of pristine Bi_2_Te_3_, while maintaining competitive Seebeck coefficients, leading to a 15% enhancement in power factor. Thermal conductivity decreased by over 50% due to the enhanced phonon scattering from nanoparticle interfaces and reduced grain size, resulting in a more than 3-fold increase in figure of merit. In addition, the films exhibited improved micromechanical properties, with hardness and elastic modulus increasing by approximately 1.25 and 2 times, respectively. These results demonstrate a scalable strategy to develop mechanically robust, high-performance thermoelectric films for microscale waste-heat energy harvesting.

## Highlights

• Carbon black nanoparticles doubled electrical conductivity of Bi_2_Te_3_ films
• Thermal conductivity reduced by >50% via phonon scattering
• Figure of merit increased by more than 3-fold
• Mechanical strength improved, with higher hardness and modulus

## Graphical abstract

**Figure:**
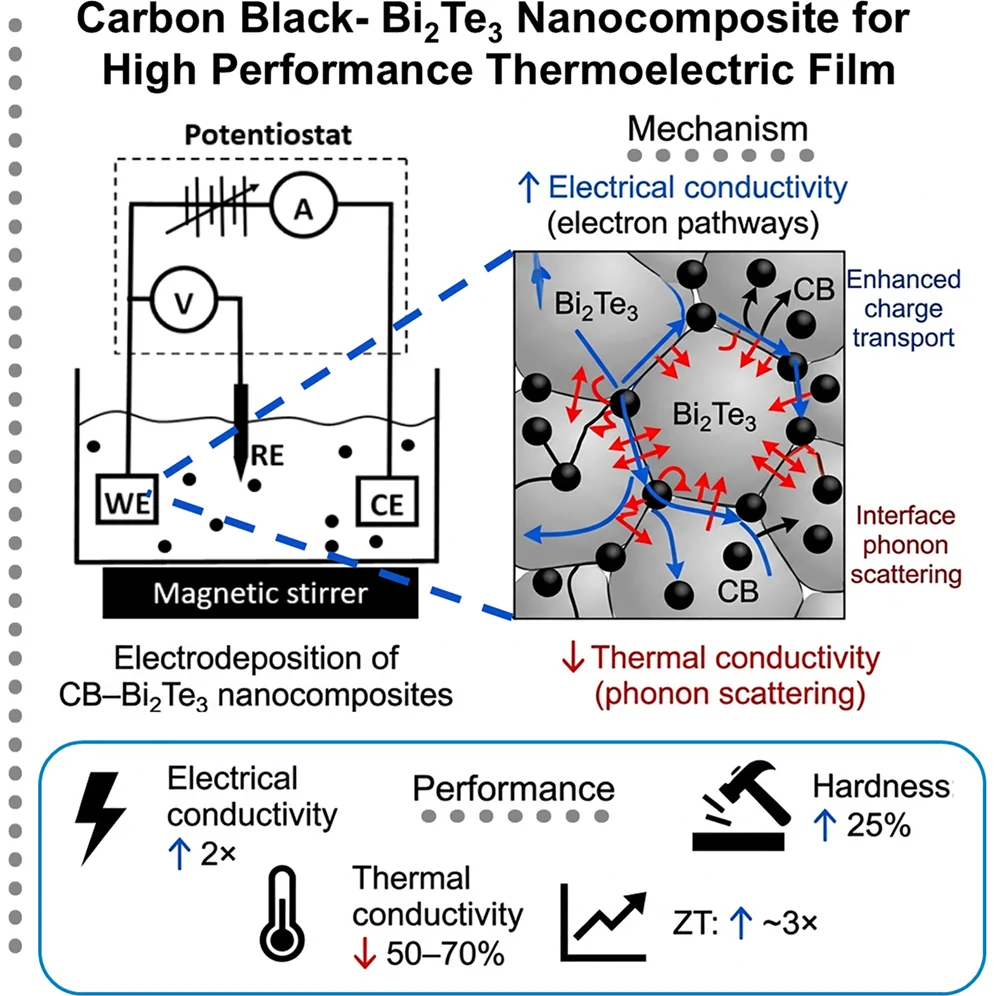

## Teaser

Physics; Engineering; Materials science

## Introduction

Thermoelectric generator (TEG) devices can produce electrical energy by utilizing surrounding waste heat. The rapid growth of energy-harvesting technologies associated with the Internet of Things (IoT) has led to increasing demand for the development of high-performance micro-TEGs (μTEGs).^1^^,^^2^ μTEGs could act as a charger to IoT device’s battery or even replace the battery due to a high ratio of output voltage to volume. The development of μTEGs relies on thermoelectric films fabricated using thin-film technologies compatible with microfabrication processes. However, thermoelectric films, particularly those synthesized by electrodeposition, often suffer from relatively low thermoelectric performance, which ultimately limits the performance of μTEG devices. This reduction in thermoelectric performance typically occurs at low temperature or near-room-temperature condition. Bulk bismuth telluride (Bi_2_Te_3_) is known to exhibit the highest overall thermoelectric performance, or figure of merit (ZT), near room temperature, with reported values of up to 0.7,^3^^,^^4^^,^^5^^,^^6^ while in the film condition, the ZT has been reported to drop to 0.16.^7^^,^^8^ The ZT value is given by the following equation:

$$ZT=\left(S^{2}\sigma /\kappa \right)T$$

where *S* is the Seebeck coefficient, *σ* is the electrical conductivity, *κ* is the thermal conductivity, and *T* is the absolute application temperature. Improving ZT, therefore, requires simultaneous enhancement of the power factor (*PF* = *S*^2^*σ*) and reduction of the thermal conductivity.

Substantial efforts have been made using various synthesis methods to improve the thermoelectric properties of Bi_2_Te_3_ films. One commonly used approach that does not involve doping or nanoinclusion is magnetron sputtering.^9^^,^^10^^,^^11^^,^^12^^,^^13^ This process allows the synthesis of a thin film of Bi_2_Te_3_ on various substrates including an insulating material. However, the process is costly, and the deposition of thick films remains challenging. Most of the results from this process are capable of increasing the power factor significantly by improving the electrical conductivity value. Kianwimol et al. proved that by controlling specific power of sputtering and maintaining the highly (015) crystal orientation, the carrier concentration and mobility of the deposited film can be optimized^9^; although the optimized electronic properties improved the electrical conductivity, the Seebeck coefficient decreased to −85.6 μV/K, which is lower than that of bulk Bi_2_Te_3_, and thermal conductivity was not reported. In general, thermoelectric materials with high electrical conductivity that do not employ doping or nanocomposite strategies tend to exhibit relatively high thermal conductivity.^14^^,^^15^

A nanocomposite approach provides an effective strategy to manipulate the thermal conductivity of a Bi_2_Te_3_ matrix without significantly compromising electrical conductivity. Carbon-based nanomaterials are particularly promising for reducing thermal conductivity while maintaining or enhancing electrical transport. Ahmad et al. used single-walled carbon nanotubes (SWCNTs) incorporated with a Bi_2_Te_3_ bulk matrix by using a powder mixing process and through the pressure-assisted high-frequency induction heated sintering (HFIHS).^16^ The SWCNTs functioned as an interconnected network at inter/*trans*-granular positions of Bi_2_Te_3_, which amplified the electrical properties. Additionally, newly formed interfaces between SWCNTs and Bi_2_Te_3_ increased phonon scattering, leading to lower lattice thermal conductivity. Similar behavior was reported for graphene-Bi_2_Te_3_ bulk nanocomposites,^17^ where uniformly dispersed graphene nanosheets enhanced electron mobility and suppressed grain growth. The resulting finer grain size increased interface-driven phonon scattering and reduced thermal conductivity.

Statistically, there are limited studies on the synthesis of Bi_2_Te_3_ nanocomposite films, especially with carbon-based nanomaterial inclusion.^18^^,^^19^ Generally, the process that is related to the synthesis of bulk Bi_2_Te_3_ is not quite compatible with the fabrication process of μTEGs. Meanwhile, deposition technologies such as flash evaporation,^20^^,^^21^ chemical vapor deposition (CVD),^22^^,^^23^^,^^24^ printing, and electrochemical deposition^25^^,^^26^^,^^27^ are favorably being used for μTEG development. Xu et al. reported that the multi-walled carbon nanotube (MWCNT)-Bi_2_Te_3_ nanocomposite film prepared by electrochemical deposition could reduce the electrical resistivity significantly as compared with the electrodeposited pure Bi_2_Te_3_ film.^28^ Advances in carbon-based nanocomposite thermoelectric films have further demonstrated the effectiveness of hybrid Bi_2_Te_3_ systems for flexible and microscale energy-harvesting applications. Chiba et al. reported flexible nanocomposite films composed of Bi_2_Te_3_ nanoplates and carbon nanotubes (CNTs) selectively separated by ultracentrifugation, achieving markedly enhanced thermoelectric performance, with the power factor improving by more than 100% compared with CNT-free Bi_2_Te_3_ films while maintaining mechanical flexibility.^29^ Similarly, Chen et al. developed flexible n-type thermoelectric films based on Bi_2_Te_3_ nanosheets integrated with a CNT network, where the formation of an electrically percolated CNT framework led to a significant enhancement in power factor (30%–50%) relative to pristine Bi_2_Te_3_ films.^30^ In addition to one-dimensional carbon nanostructures, two-dimensional carbon materials have also been explored. Du et al. investigated reduced graphene oxide-Bi_2_Te_3_ nanocomposites and demonstrated that the incorporation of graphene derivatives resulted in a notable reduction in thermal conductivity (on the order of 20%–40%), mainly attributed to enhanced phonon scattering at the heterogeneous interfaces, while maintaining favorable electrical transport properties.^31^ Another study reported the incorporation of diamond-like carbon (DLC) into (Bi,Sb)_2_Te_3_ films, which resulted in a slight decrease in electrical conductivity but a more than 2-fold enhancement in the Seebeck coefficient compared with pristine (Bi,Sb)_2_Te_3_ films. The films were synthesized using a pulsed laser deposition (PLD) technique.^32^

This work investigates the incorporation of porous carbon black nanoparticles (CB-NPs) into Bi_2_Te_3_ films synthesized by electrochemical deposition. High electrical conductivity properties of CB-NPs might be able to initiate the increment of electrical transport of the Bi_2_Te_3_ film. The inclusion of CB-NPs in materials can also be expected to reduce thermal conductivity by enhancing phonon scattering, as proven by previous studies that involved carbon-based nanomaterials.^16^^,^^33^ In particular, no study has analyzed the incorporation of CB-NPs in Bi_2_Te_3_ films. The closest related study was in bulk Bi_2_Te_3_ condition with 1.0 wt % CB-NPs. The bulk nanocomposite was synthesized by pressing the blended powders. Then, the compacted sample was sintered under specific condition.^34^ The study found a very good result on the overall thermoelectric efficiency where more than twice the value was recorded for the sample as compared with pure bulk Bi_2_Te_3_. The increased performance is mainly attributed to lower thermal diffusivity that generally results in lower thermal conductivity. However, only a low CB content was investigated, and the influence of different CB concentrations in the Bi₂Te₃ matrix was not explored, limiting the assessment of CB nanoparticle incorporation on the thermoelectric performance of Bi_2_Te_3_. In addition to thermoelectric performance considerations, CB-NPs offer practical advantages compared with CNTs and graphene, including lower material cost, higher production scalability, and reduced synthesis complexity. These features make CB-based systems more attractive for sustainable thin-film fabrication and large-area μTEG production, particularly when scalable electrodeposition processes are employed. This study, therefore, set out to assess the thermoelectric performance effect of several percentages (>3.0 wt %) of porous CB-NPs incorporated in the Bi_2_Te_3_ film. For comparison, the effect of CNT nanoinclusion on the power factor is examined. In addition, the mechanical properties of the nanocomposite films, including hardness and Young’s modulus, are evaluated relative to pristine Bi_2_Te_3_ films.

## Results and discussion

### Composition and morphological analysis

The elemental composition in deposited CB-Bi_2_Te_3_ nanocomposite films with respective electrolytes is summarized in Table 1. The amount of co-deposited CB-NPs in the nanocomposite film was estimated as carbon wt % recorded from the average values of energy-dispersive X-ray (EDX) measurements. The deposited films were prepared with a carbon concentration up to 4.3 wt % by referring to the optimized condition of the measured thermoelectric performances (detailed discussion in section evaluation of thermoelectric power factor). The atomic ratio of Bi/Te with the inclusion of CB-NPs was not affected significantly as compared with the typical pure Bi_2_Te_3_ phase ratio, with the percentage of variation maintained within no more than ±2.0%. Generally (in the case of pure Bi_2_Te_3_), with such a low percentage of error, the electrodeposited Bi_2_Te_3_ films do not show significant differences in terms of the thermoelectric performance.^35^^,^^36^^,^^37^ EDX elemental mapping reveals a uniform spatial distribution of bismuth, tellurium, and carbon throughout the CB-Bi_2_Te_3_ films, indicating successful and well-balanced incorporation of CB-NPs into the Bi_2_Te_3_ matrix. The corresponding elemental maps (Data S1, Figure S1-1) show that carbon signals are evenly dispersed across the mapped area without localized clustering, suggesting that CB-NP aggregation is effectively suppressed during deposition. Such homogeneous dispersion of CB-NPs introduces a high density of nanoscale heterointerfaces within the film, which are effective sites for phonon scattering. As a result, the well-distributed CB-NPs are expected to limit phonon propagation through enhanced interface, impurity, and defect-related scattering, thereby contributing to the observed reduction in lattice thermal conductivity.

**Table 1:** CB concentration in electrolyte and element composition of electrodeposited films

Electrolytes	CB content in electrolyte (g/L)	Electrodeposited films	Composition in the deposited films			
			C (wt %)	Bi:Te (atom %)	Non-stoichiometric compounds of Bi/Te	Atomic percentage error due to Bi_2_Te_3_ phase ratio (%)
I	0.000	Bi_2_Te_3_	0.0	40:60	Bi_2__.__00_Te_3.00_	0
II	0.020	CB-Bi_2_Te_3_-I	3.4	42:58	Bi_2__.__10_Te_2.90_	±2
III	0.025	CB-Bi_2_Te_3_-II	3.7	41:59	Bi_2__.__05_Te_2.95_	±1
IV	0.035	CB-Bi_2_Te_3_-III	4.3	41:59	Bi_2__.__05_Te_2.95_	±1

**Figure 1:**
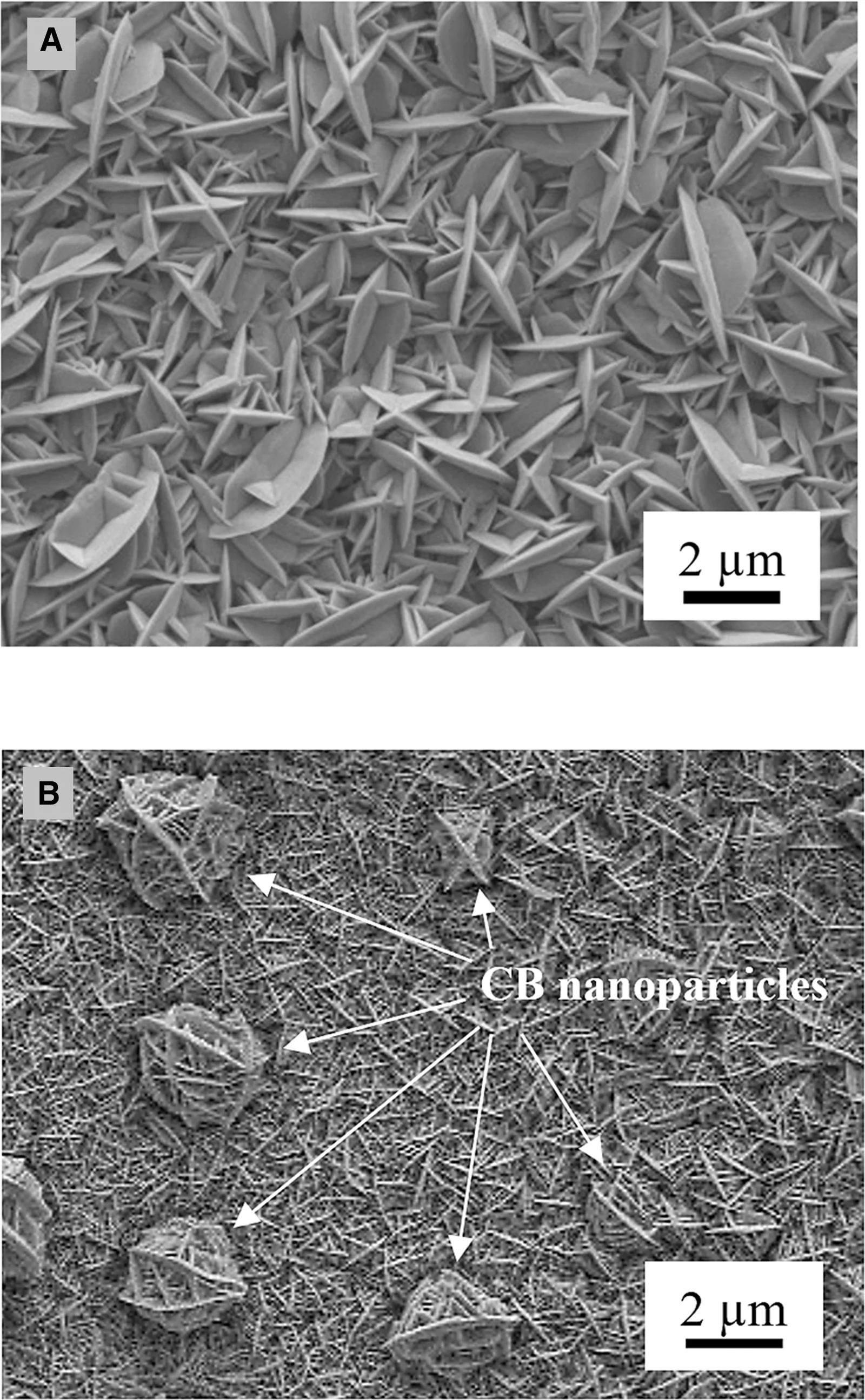
SEM micrograph images of the electrodeposited film surface (A) Pure Bi_2_Te_3_. (B) CB-Bi_2_Te_3_. The CB-NPs were covered by the growth of Bi_2_Te_3_ due to adequate electrically conductive condition. Scale bars shown in the images correspond to 2 μm.

Typical growth of the electrodeposited Bi_2_Te_3_ surface-crystal structure, which is known as the plate-like structure, is shown in Figure 1A. Figure 1B shows that the microcrystal growth on CB-Bi_2_Te_3_ also had identical crystal shape as pure Bi_2_Te_3_; however, the size was quite smaller. It can clearly be seen that the CB-NPs deposited on the nanocomposite film, with the growth of the Bi_2_Te_3_ microstructures around the NPs. The deposition of CB-NPs was likely to suppress the crystal growth due to polymeric coating around the particle. If only free polymeric coated CB-NPs co-deposited in Bi_2_Te_3_, the reduction of Bi/Te ions can be expected to become faster. This is due to the higher-efficiency electron transfer occurred by the capability of CB-NPs to act as a conductive catalyzer during electro-co-deposition process.^38^ Besides that, the existence of poly(diallyldimethylammonium chloride) (PDDA)-coated CB-NPs in electrolyte made the electron transfer to Bi/Te ions less efficient during the deposition and finally reduced the Bi_2_Te_3_ crystals growth. The slower rate of the crystal growth can be confirmed by different deposition rates recorded on the pure Bi_2_Te_3_ and CB-Bi_2_Te_3_ films. With the same set of electrodeposition parameters, roughly the nanocomposite deposition rate takes approximately 40% slower than the pristine film.

**Figure 2:**
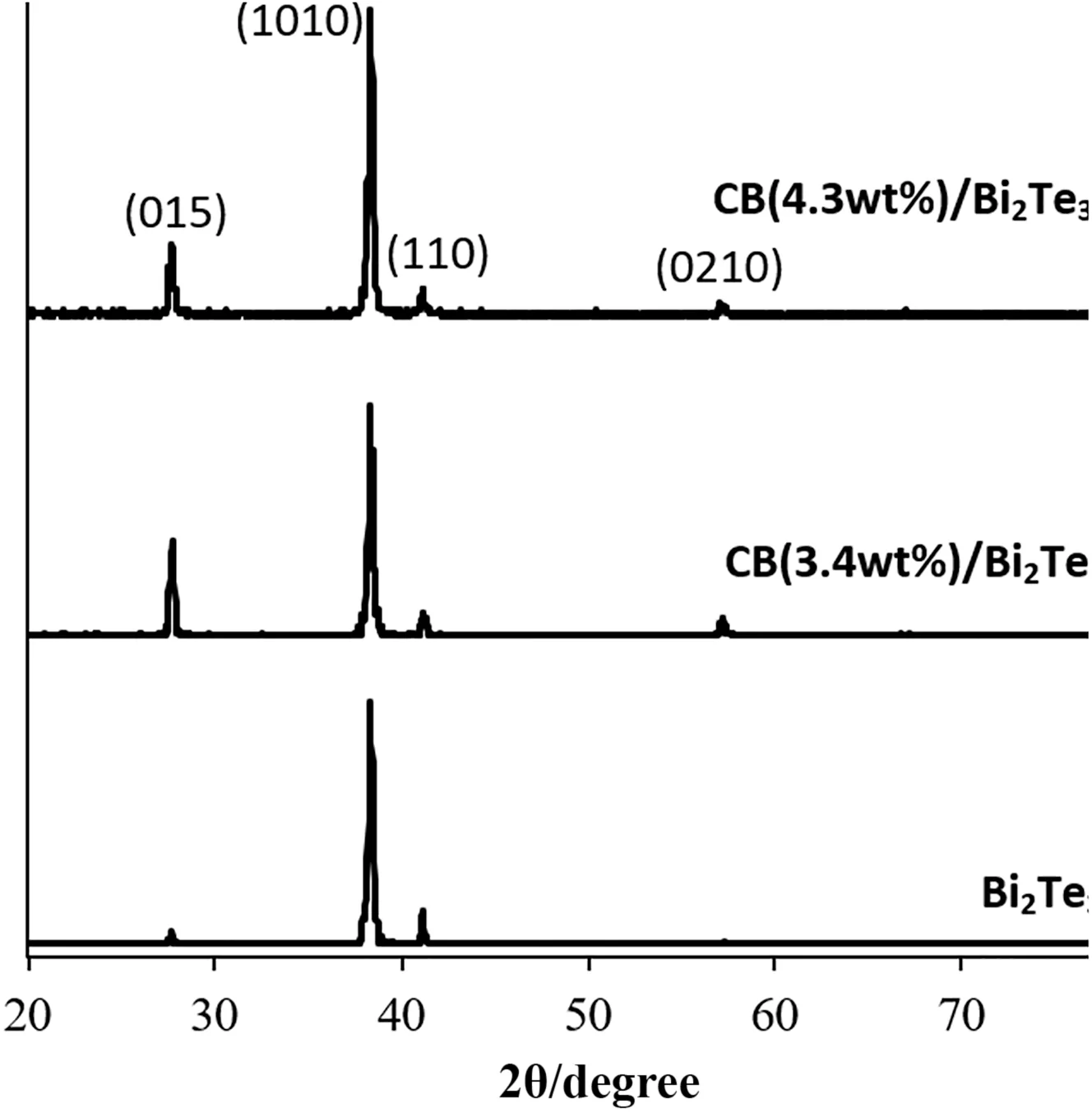
XRD data for pure Bi_2_Te_3_ and CB-Bi_2_Te_3_ at 3.4 wt % and 4.3 wt % of CB-NPs deposited

Figure 2 depicts X-ray diffraction (XRD) pattern of the pure Bi_2_Te_3_ and the nanocomposite films. The presented data of nanocomposite films are available only for the minimum and maximum amounts of the CB inclusion in the nanocomposite films. The significant diffraction peaks detected from all samples were crystal planes of (015), (1010), (110), and (0210), which are in good agreement with that of the electrodeposited pristine Bi_2_Te_3_ reported in previous studies.^7^^,^^39^^,^^40^^,^^41^ The electrodeposited Bi_2_Te_3_ and CB-Bi_2_Te_3_ films were formed as polycrystalline oriented structures, with a dominant peak at (1010). The (1010) orientation was quite a typical orientation for the electrodeposition using negative working electrode potentials. The nanocomposite films exhibit a stronger (015) orientation peak than the pristine film. Generally, with the existence of (015) orientation, the film is likely to have a rougher surface and courser surface structure that lead to low-density formation.^41^ However, the characteristic was on the contrary to this study where the smaller surface-microstructure was grown for the nanocomposite film, and the influence of (015) orientation toward characteristic of the film morphology can be changed with different deposition rates. Zhou et al. performed a detailed study on the electrodeposited Bi_2_Te_3_ films by changing mainly the duty cycle of electrodeposition, resulting in different deposition rates.^7^ A lower deposition rate has been found to not only result in a smaller grain size but also reduce the surface roughness. Similarly, in this study, even when the (015) orientation was detected, the nanocomposite film was grown with more compact and higher density. The average grain size of the nanocomposites decreased, as summarized in Table 2. The grain size was approximately 31 nm, which is about 12% smaller than that of pristine Bi_2_Te_3_.

**Table 2:** Summary of the average grain size of pristine Bi_2_Te_3_ films and CB-Bi_2_Te_3_ nanocomposite films, determined from XRD peaks at 2*θ* = 27.7°, 38.3°, and 41.2°

Film type	Integral breadth, *β* at *2θ =38.3*, (rad)	Average grain size (nm)
Bi_2_Te_3_	4.80 × 10^−3^	34.7 ± 1.0
CB (3.4 wt %)-Bi_2_Te_3_	5.11 × 10^−3^	30.6 ± 1.3
CB (3.7 wt %)-Bi_2_Te_3_	5.07 × 10^−3^	30.5 ± 1.4
CB (4.3 wt %)-Bi_2_Te_3_	5.21 × 10^−3^	30.8 ± 1.0

### Evaluation of thermoelectric power factor

**Figure 3:**
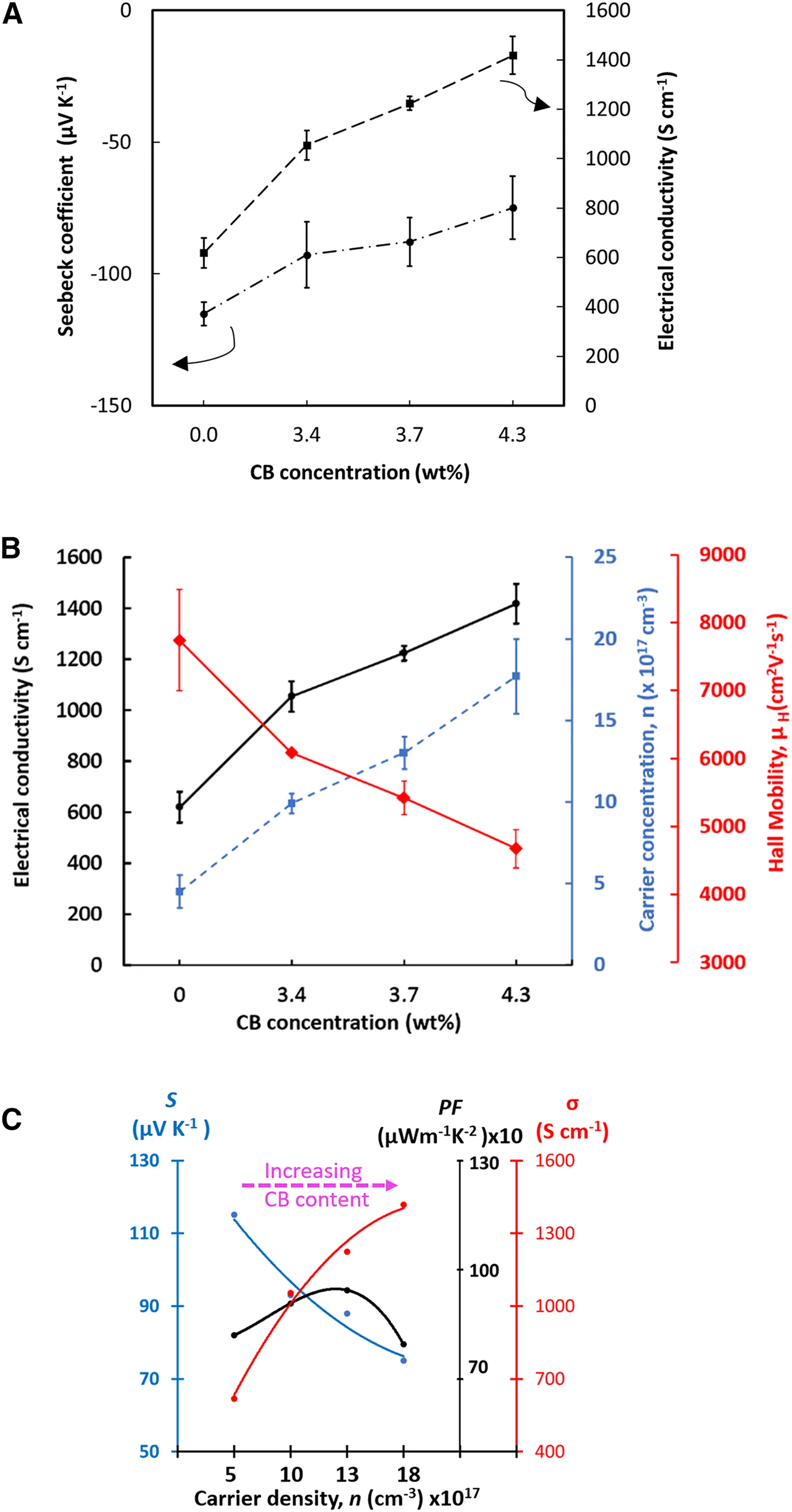
Dependences of Seebeck coefficient, electrical conductivity, and carrier concentration on the deposited CB-NP concentration (A) Seebeck coefficient dependence on the deposited CB-NP concentration. (B) Electrical conductivity dependence on the deposited CB-NP concentration. (C) Carrier concentration dependence on the deposited CB-NP concentration. *S*, Seebeck coefficient; σ, electrical conductivity; *PF*, power factor, with respect to the carrier concentration. Data in (A) and (B) are represented as mean values, with error bars indicating the minimum–maximum range.

Figure 3A depicts the results of thermoelectric performances, specifically in terms of the dependence of Seebeck coefficient and electrical conductivity on the CB-NP concentration. The inclusion of CB-NPs significantly increased the electrical conductivity by up to more than two times, at 4.3 wt % of carbon, compared with that of the pristine Bi_2_Te_3_ film. A steady trend of improvement in the electrical conductivity could be found as the amount of CB-NP inclusion increased in the nanocomposite films. Generally, semiconductor materials with a high electrical conductivity possess a high carrier concentration. In line with the results shown in Figure 3B, as the deposited amount of CB-NPs in the nanocomposite increased, the carrier concentration also gradually increased. With CB-NPs’ naturally high electrical conductive capability, the effect of the carrier contribution (specifically on electron concentration) to the N-type-based nanocomposite can be quite identical to those of metal and semi-metal NPs.^42^^,^^43^ Because all the measurements were made in a low-temperature environment, CB-NPs had notable effects on scattering of the low-energy electrons, rather than that of the high-energy ones. In contrast, the Hall mobility decreases from 8,581.6 to 5,001.4 cm^2^ V^−1^ s^−1^ with increasing CB content, as shown as shown in Figure 3B, indicating enhanced carrier scattering due to additional interfaces and defects introduced by CB incorporation. Similar trade-offs between increased carrier concentration and reduced mobility have been reported in Bi_2_Te_3_ systems containing CNTs or graphene, where interface scattering accompanies carrier injection.^15^^,^^16^^,^^17^ The absence of mobility enhancement suggests that CB-NPs do not form a continuous percolated conductive network; instead, they act as dispersed conductive inclusions that increase electron availability while introducing moderate scattering. Overall, the net increase in electrical conductivity arises from the dominance of carrier concentration enhancement over mobility reduction, highlighting a carrier density-driven transport mechanism in the CB-Bi_2_Te_3_ films, as discussed by Mona et al.^44^

Meanwhile, the Seebeck coefficient values for the nanocomposite films reduced as the CB-NP concentration increased. The reduced value of Seebeck coefficient was indirectly proportional to the increment of the electrical conductivity. The lower Seebeck coefficient was influenced by a higher carrier concentration, as reported in previous studies.^43^^,^^45^^,^^46^ The measured carrier concentration of the nanocomposite film consistently proved that a higher carrier concentration contributes to a lower Seebeck coefficient, as shown in Figure 3C. The inverse effect was identical with the following expression^47^:

$$S=\left(8\pi^{2}k_{B}^{2}/3eh^{2}\right)m^{*}T{\left(\pi /3n\right)}^{2/3}\text{,}$$

where *k*_*B*_*, h, e, n*, and *m∗* are Boltzmann’s constant, Planck’s constant, electronic charge, carrier concentration, and effective mass of the carrier, respectively. The equation strongly proposed that a thermoelectric material with a higher carrier density has a lower absolute value of the Seebeck coefficient. Figure 3C also shows the calculated values on power factor (*PF* = *S*^2^*σ*); the highest value was found at 3.7 wt % for deposited CB-NPs, and it was largely influenced by the second highest value of the electrical conductivity.

**Figure 4:**
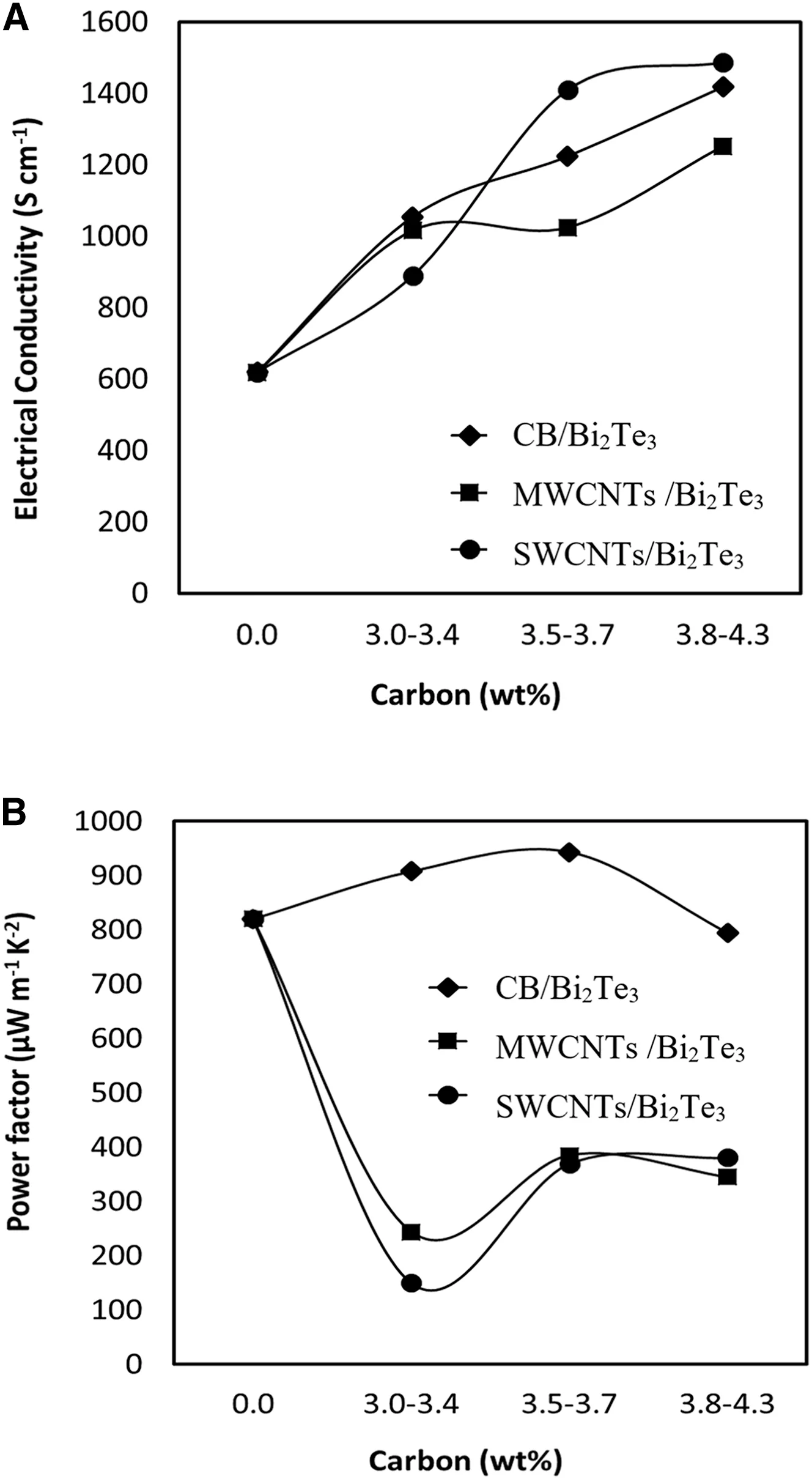
Electrical conductivity and power factor dependences on the deposited CB-NP (represented as carbon wt %), MWCNT, and SWCNT concentrations (A) Electrical conductivity dependence. (B) Power factor dependence.

This study also performed a comparison between CB-Bi_2_Te_3_ and CNT-Bi_2_Te_3_ films by evaluating the power factor. CB-Bi₂Te₃ and CNT-Bi₂Te₃ films with comparable carbon contents were used for the comparison, with the difference in carbon loading maintained within ±0.3 wt.% of incorporated CB-NPs or CNTs. The methods of preparation and synthesis for both types of CNT inclusion in the Bi_2_Te_3_ film were similar as for the CB-NPs. Figure 4 shows a comparison data of electrical conductivity and power factor dependences on different inclusion of CB-NPs, MWCNTs, and SWCNTs. Inclusion of all of the nanomaterials contributed to the significant increment in the electrical conductivity, with the maximum value recorded at 4.3 wt %. Starting at 3.4 wt % and above, the SWCNT inclusion in Bi_2_Te_3_ film influenced the electrical conductivity the most as compared with other nanocomposite films, with the maximum value being 140% higher than that of pure Bi_2_Te_3_. It is worth to mention that, at minimum 3.8 wt % of carbon, all the carbon-based nanomaterials effectively increased the electrical conductivity by at least double the value of the pure Bi_2_Te_3_ film.

The electrical conductivity of the CB-Bi_2_Te_3_ films increases monotonically with the CB content, rising from 618.6 S cm^−1^ for pristine Bi_2_Te_3_ to 1,418.2 S cm^−1^ at 4.3 wt % CB, which closely follows the increase in carrier concentration from 4.5 × 10^19^ to 17.7 × 10^19^ cm^−3^ as shown in Figure 3B. This trend indicates that the conductivity enhancement is primarily driven by carrier density modulation, rather than mobility improvement. The observed increase in carrier concentration suggests a doping-like effect associated with the CB-NPs, likely originating from charge transfer at CB-Bi_2_Te_3_ interfaces, consistent with the carrier modulation mechanisms reported for carbon-based thermoelectric nanocomposites. In contrast, the Hall mobility decreases from 8581.6 to 5001.4 cm^2^ V^−1^ s^−1^ with the increasing CB content, indicating enhanced carrier scattering due to additional interfaces and defects introduced by CB incorporation. Similar trade-offs between increased carrier concentration and reduced mobility have been reported in Bi_2_Te_3_ systems containing CNTs or graphene, where interface scattering accompanies carrier injection.^15^^,^^16^^,^^17^ The absence of mobility enhancement suggests that CB-NPs do not form a continuous percolated conductive network; instead, they act as dispersed conductive inclusions that increase electron availability while introducing moderate scattering. Overall, the net increase in electrical conductivity arises from the dominance of carrier concentration enhancement over mobility reduction, highlighting a carrier-density-driven transport mechanism in the CB-Bi_2_Te_3_ films.

Both SCWCNT and MWCNT nanocomposite films exhibited a significant trade-off between electrical conductivity and Seebeck coefficient. The trade-off led to a notable reduction in the power factor, as can be seen in Figure 4B. Interestingly, the power factor for CNT-Bi_2_Te_3_ films had an insignificant difference when the carbon content reached 3.5 wt % and above. Compared with pure Bi2Te3, the power factor values declined by more than half. In contrast, the power factor of the CB-Bi_2_Te_3_ films increased by up to 15% at a range of 3.5–3.7 wt % of carbon as compared with pure Bi_2_Te_3_ and then started to decline when the carbon wt % was more than 3.7. Higher power factor values recorded on the CB-Bi_2_Te_3_ films were prominently contributed from adequate values of the Seebeck coefficient. Even though the Seebeck coefficient of CB-Bi_2_Te_3_ films experienced a drop of up to 35%, the CNT-Bi2Te3 films exhibited a substantial loss in values, dropping to −51 μV/K, which represents a decrease of almost 56%. A huge loss on the Seebeck coefficient values leads to the very low power factor on both CNT-Bi_2_Te_3_ films.

### Evaluation of thermal conductivity and ZT

**Figure 5:**
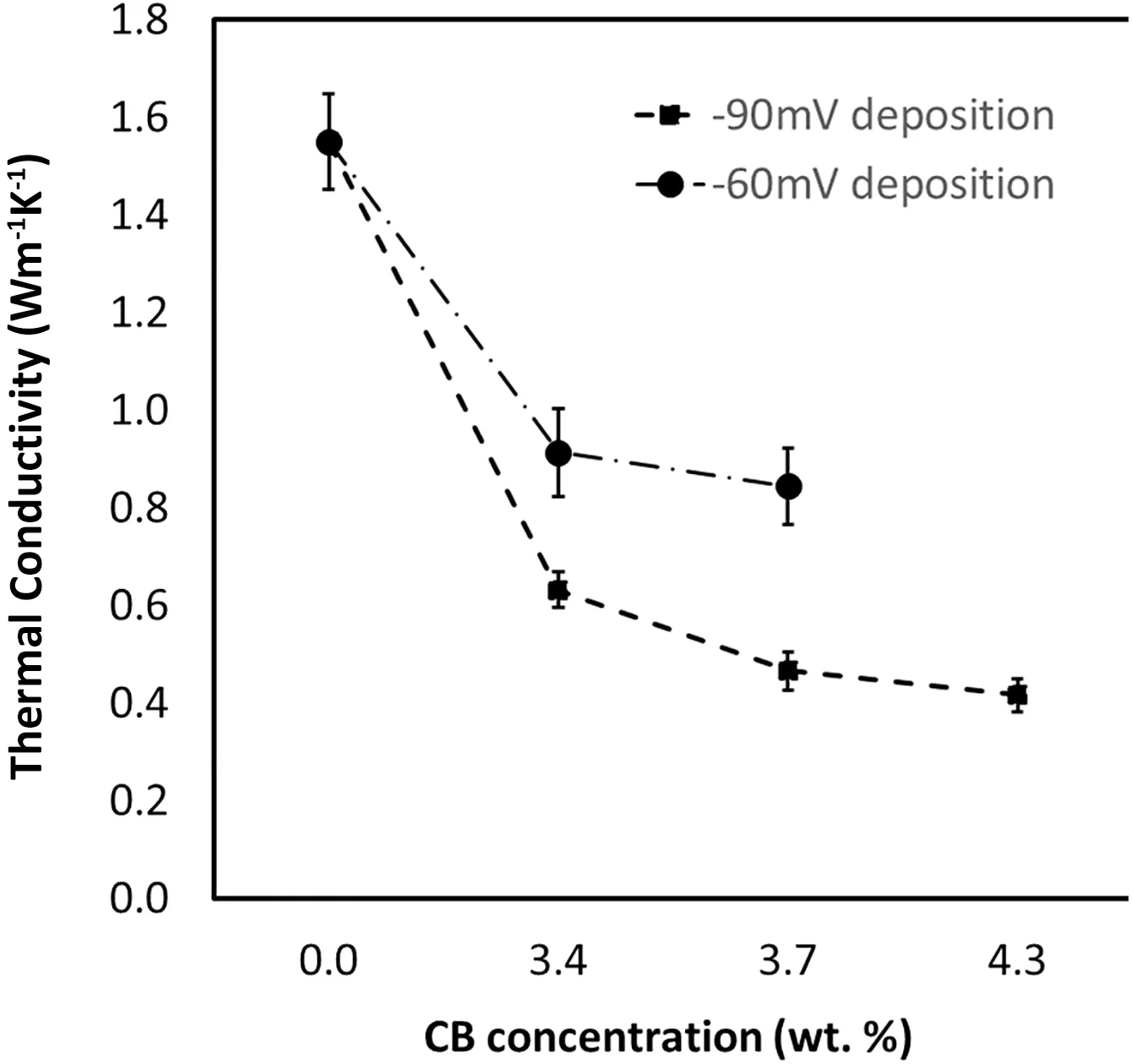
Deposited CB-NP concentration dependence of thermal conductivity at electro co-deposition potentials of −60 and −90 mV Data are represented as mean values, with error bars indicating the minimum–maximum range.

Figure 5 shows the thermoelectric performances in terms of the thermal conductivity for the CB-Bi_2_Te_3_ nanocomposite films at −60 and −90 mV potentials for electro co-deposition. This study used −90 mV of working electrode potential as a non-variable parameter in the electrodeposition process to evaluate the influence of co-deposited CB-NPs in the nanocomposite films. An attempt has been made to deposit using −60 mV to investigate the effect on the thermal conductivity performance. Generally, both electrodeposition potentials lead to lower thermal conductivity than that for the pristine Bi_2_Te_3_; however, higher electrodeposition potential produces even lower thermal conductivity, which is below 0.7 Wm^−1^ K^−1^. The lower value can be expected due to possible smaller grain size attributed to the film. It is a typical phenomenon on the electrodeposited Bi_2_Te_3_ film; when a higher negative potential is applied, the grain size becomes smaller.^41^ Smaller grain size promotes more grain boundaries and significantly increases the phonon scattering. The inclusion of CB-NPs in the Bi_2_Te_3_ films resulted in a significant reduction in thermal conductivity, beginning at a CB content of 3.4 wt % and steadily decreasing as the concentration of CB-NPs increased. The lowest thermal conductivity recorded was 0.4 at 4.3 wt % CB concentration, which plummeted 73% as compared with that of the pristine Bi_2_Te_3_ film.

The total thermal conductivity was not experimentally separated into electronic (κ_e_) and lattice (κ_l_) contributions in this study. Nevertheless, insight into the dominant transport mechanism can be inferred from the measured electrical properties. With the increasing CB-NP content, the electrical conductivity increases significantly, which would be expected to increase κ_e_ according to the Wiedemann-Franz relationship. In contrast, the measured total thermal conductivity exhibits a substantial reduction of up to 73%, indicating that the decrease cannot be attributed to changes in electronic thermal transport alone. Furthermore, the monotonic decrease in Hall mobility with the increasing CB-NP concentration implies enhanced carrier scattering due to additional interfaces, defects, and grain boundaries introduced by CB incorporation. These features are also effective phonon-scattering centers, supporting a lattice-dominated thermal transport mechanism.^44^ Combined with the observed reduction in grain size and the presence of porous CB-NPs, which promote impurity and boundary phonon scattering, the results strongly indicate that κ_l_ plays a decisive role in the observed suppression of thermal conductivity.^48^

In general, lattice thermal conductivity is governed by the phonon mean free path, and any mechanism that enhances phonon scattering will effectively reduce this mean free path and consequently suppress heat transport. The effective mean free path, *l*_*eff*_, is theoretically related to phonon scattering, which can be stated based on Matthiessen’s relationship,^45^^,^^46^

$$\frac{1}{l_{eff}}=\frac{1}{l_{b}}+\frac{1}{l_{i}}+\frac{1}{l_{p}}\text{,}$$

where *l*_*b*_, *l*_*i*_, and *l*_*p*_ are mean free path of the boundary, impurity or defect scattering, and intrinsic phonon-phonon interactions, respectively. The long-wavelength phonon was dominant in thermal transportation at room temperature condition according to Wien’s displacement law for phonon states.^49^ The long-wavelength phonon for the nanocomposite films was effectively scattered by the CB-NPs and increased the impurity scattering, *l*_*i*_. The similar effect has been reported in recent studies of the Bi_2_Te_3_-based film blended with other type of NPs.^50^^,^^51^ In addition, the porous characteristic of CB-NPs promotes lower impurity mass and increases the localized phonon scattering.^52^^,^^53^ As film porosity is reflected in its density, density measurements indicate only minor variations across all samples (<3%), with no discernible correlation between density and the observed reduction in thermal conductivity.

**Figure 6:**
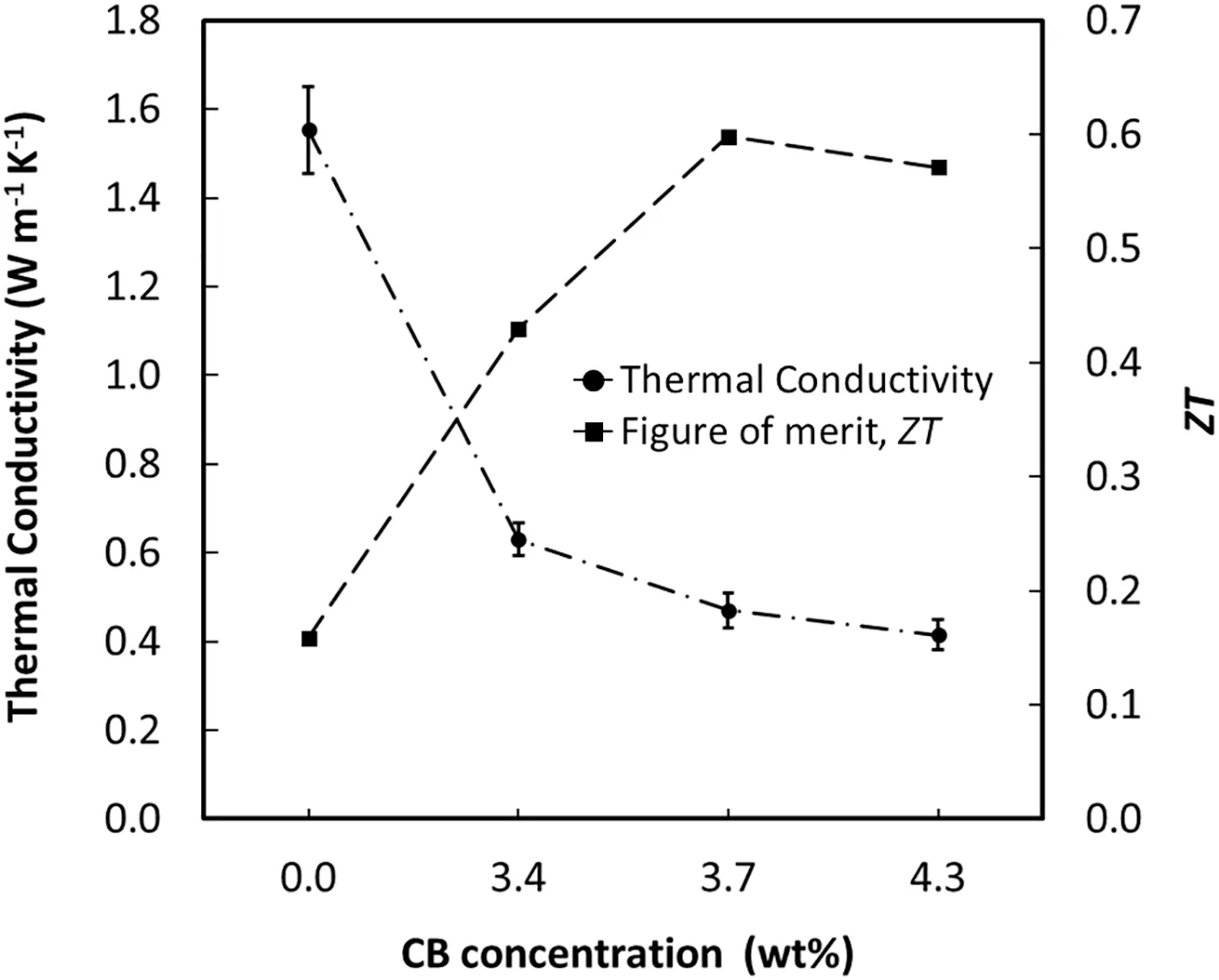
Deposited CB-NP concentration dependences of thermal conductivity and ZT Data are represented as mean values, with error bars indicating the minimum–maximum range.

The long-wavelength phonon was also effectively scattered by grain boundary. Smaller crystallite sizes in the nanocomposite films contribute to a higher concentration of finer grain sizes, as discussed earlier. A finer grain significantly reduced thermal conductivity due to a higher number of grain boundaries, as found in previous studies.^54^^,^^55^ Even though heat transfer through the short-wavelength phonon is not dominant, a significantly suppressed phonon might have contributed to reduce the total thermal conductivity. Figure 6 demonstrates the correlation between decreased thermal conductivity values and enhanced ZT for CB-Bi_2_Te_3_ films. Overall, the ZT values were amplified as the CB-NP concentration increased. The maximum ZT obtained was 0.6, which was almost triple that of the pristine film (optimized at 3.7 wt % of CB-NPs). The ZT value on 4.3 wt % of CB-NPs was slightly declined because of the significant drop in power factor.

### Evaluation of micromechanical properties

**Figure 7:**
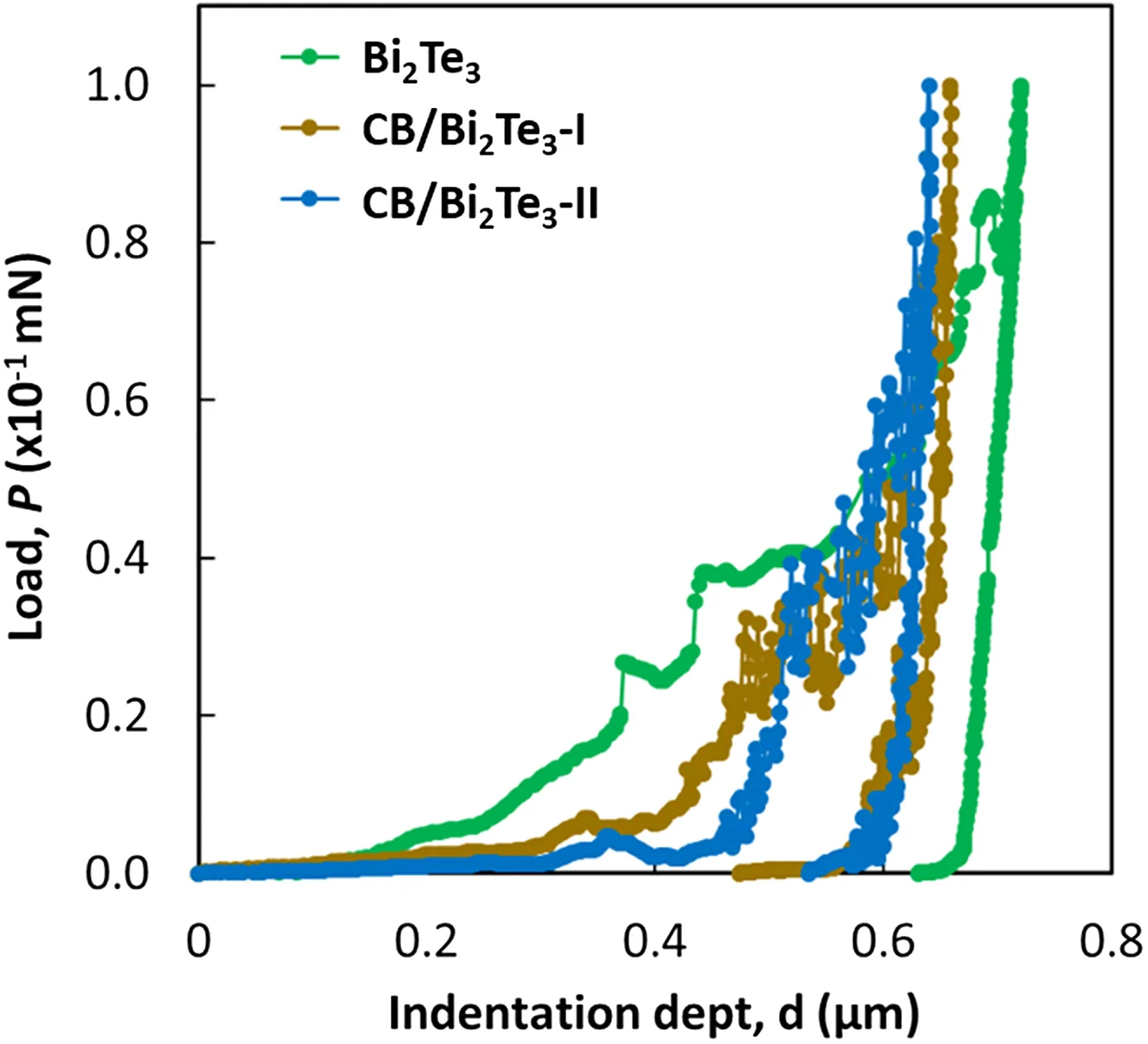
Indentation curves for pristine Bi_2_Te_3_ and CB-Bi_2_Te_3_-I, and CB-Bi_2_Te_3_-II nanocomposite films

The micromechanical properties of two samples of CB-Bi_2_Te_3_ films were compared with those of pristine Bi_2_Te_3_, which contains 4.3 wt % of CB-NPs (CB-Bi_2_Te_3_-I) and a higher percent of CB-NP content at 5.5 wt % (CB-Bi_2_Te_3_-II). An additional CB-Bi₂Te₃ sample with a higher CB-NP content of 5.5 wt.% was prepared to further evaluate the effect of increased CB incorporation on the micromechanical strengthening of the Bi₂Te₃ film. The hardness value was calculated based on the measured indentation depth and its corresponding load, as shown in Figure 7 *d*_*1*_ and *d*_*2*_ present the indentation depth in plastic deformation of the Bi_2_Te_3_ and CB-Bi_2_Te_3_ samples. From the indentation perspective, the material with higher hardness value was capable of hindering the plastic deformation better than the one with lower hardness. The plastic deformation of CB-Bi_2_Te_3,_ *d*_*2*_, was larger than that of Bi_2_Te_3_, which indicated that the hardness value is larger. The measured hardness values of the pristine electrodeposited Bi_2_Te_3_ in this study were compared with the prior finding of electrodeposited pure Bi_2_Te_3_ film, and both values were about 0.4 GPa.^56^ The hardness values of both CB-Bi_2_Te_3_ films were in good agreement, with no significant difference. Overall, the hardness value of CB-Bi_2_Te_3_ increased by approximately 25% compared with the pristine Bi_2_Te_3_ film.

**Figure 8:**
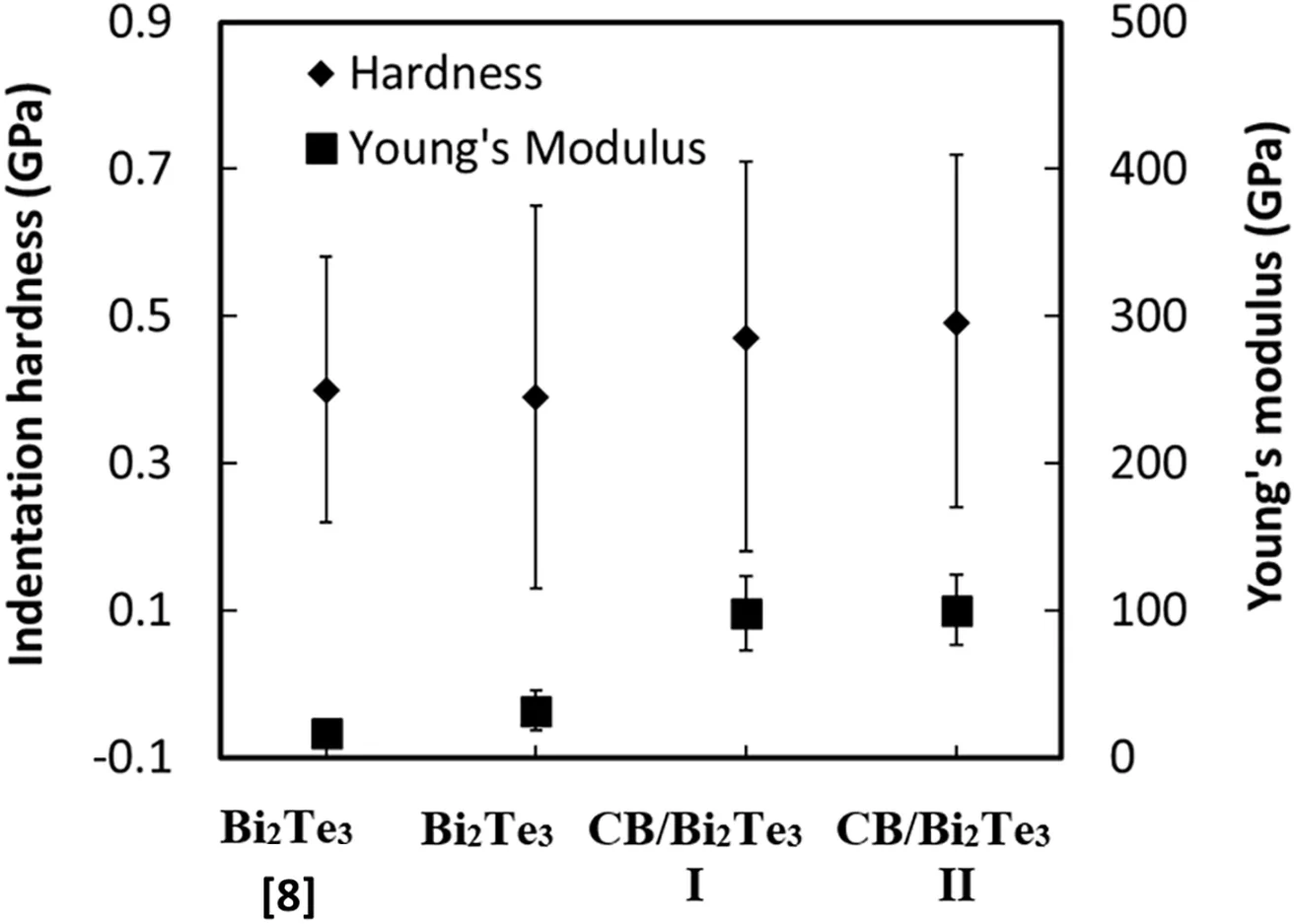
Measured hardnesses and estimated Young’s modulus of previously reported Bi_2_Te_3_ film,Bi_2_Te_3_ film from the present work, and the CB-Bi_2_Te_3_ nanocomposite films Data are represented as mean values, with error bars indicating the minimum–maximum range.

Figure 8 shows the comparison of the measured hardnesses and the estimated elastic moduli of the pure Bi_2_Te_3_ and the CB-Bi_2_Te_3_ nanocomposite films. The obtained value of the Young’s modulus for the nanocomposite shows a significant improvement, which is more than twice as compared with the pure one. The elastic modulus was estimated based on the value of hardness and the tangent of elastic recovery (unloading curve), which followed Doerner-Nix method.^57^ The enhancement in hardness and Young’s modulus observed in the CB-Bi_2_Te_3_ nanocomposite films can be attributed to multiple reinforcing mechanisms associated with the incorporation of CB-NPs. The non-covalent surface modification of CB-NPs with a polymeric coating improves interfacial adhesion between the CB-NPs and the Bi_2_Te_3_ matrix, facilitating effective load transfer across the interface under mechanical deformation.^58^ Stronger interfacial bonding suppresses interfacial debonding and limits the localized plastic deformation, thereby contributing to increased stiffness and hardness.

In addition, the presence of CB-NPs at grain boundaries and within the Bi_2_Te_3_ matrix impedes dislocation motion through a particle-pinning mechanism. CB-NPs act as physical obstacles that obstruct dislocation glide, increasing the stress required for plastic deformation. This effect is further enhanced by the fine grain structure of the nanocomposite films, where a higher density of grain boundaries provides additional barriers to the dislocation movement. As a result, dislocation accumulation and pile-up are delayed, leading to improved resistance against deformation.^59^ Moreover, the uniform dispersion of CB-NPs within the Bi_2_Te_3_ matrix, as confirmed by microstructural and elemental analyses, promotes a homogeneous distribution of stress during indentation. This reduces strain localization and inhibits crack initiation at grain boundaries, which further enhances the elastic modulus and hardness. Similar strengthening behavior has been reported in NP-reinforced composite systems, where finely dispersed secondary phases effectively restrict the dislocation activity and improve the mechanical integrity.^7^ Collectively, these mechanisms enhanced interfacial adhesion, dislocation pinning, and grain-boundary strengthening effect for the observed improvements in hardness and Young’s modulus of the CB-Bi_2_Te_3_ nanocomposite films.

### Limitations of the study

While this study demonstrates significant enhancement in the thermoelectric and micromechanical performance of CB-Bi_2_Te_3_ nanocomposite films, several limitations should be noted. First, the thermoelectric evaluation was conducted primarily at room temperature, and the temperature-dependent behavior of the materials was not investigated. As thermoelectric performance is highly sensitive to temperature, further studies across a broader operating range are required to assess practical applicability. Second, the total thermal conductivity was measured without separating κ_e_ and κ_l_ contributions. Although indirect analysis suggests that lattice thermal transport dominates the observed reduction, direct quantification would provide deeper insight into the phonon transport mechanisms. Third, the dispersion and interfacial characteristics of CB-NPs were inferred from scanning electron microscopy (SEM) and EDX analyses, but high-resolution nanoscale interfacial characterization remains limited. Advanced techniques could further clarify the role of interfaces in carrier and phonon scattering. Finally, long-term stability, environmental durability, and device-level integration of the nanocomposite films were not examined. Evaluating these aspects is essential for practical deployment of such films in μTEG systems.

## Resource availability

### Lead contact

Requests for further information and resources should be directed to and will be fulfilled by the lead contact, Khairul Fadzli (khairul.fadzli@utem.edu.my).

### Materials availability

This study did not generate new unique reagents.

### Data and code availability

• Data: All data reported in this paper will be shared by the lead contact upon request.
• Code: This paper does not report original code.
• Additional information: Any additional information required to reanalyze the data reported in this paper is available from the lead contact upon request.

## Acknowledgments

This work was mainly performed in the Micro/Nanomachining Research Education Center (MNC) of Tohoku University and supported by the 10.13039/501100009538Council for Science, Technology and Innovation, Cross-ministerial Strategic Innovation Promotion Program (SIP), Japan (The New Energy and Industrial Technology Development Organization, NEDO). One of the authors, Khairul Fadzli Samat, would like to express his gratitude to 10.13039/501100004768Universiti Teknikal Malaysia Melaka and the 10.13039/501100003093Ministry of Higher Education, Malaysia (through the Fundamental Research Grant Scheme (FRGS), no. FRGS/1/2023/TK08/UTeM/02/5) for their support in this study.

## Author contributions

Conceptualization, T.O.; methodology, T.O. and K.F.S.; investigation, K.F.S. and N.V.T.; writing – original draft, K.F.S.; writing – review & editing, K.F.S., M.A.A., M.A.I.C.A., M.H.J., and T.O.; resources, N.V.T.; supervision, T.O.

## Declaration of interests

The authors declare no competing interests.

## STAR★Methods

### Key resources table

**Table:** 

REAGENT or RESOURCE	SOURCE	IDENTIFIER
**Chemicals, peptides, and recombinant proteins**		

Bismuth oxide (Bi_2_O_3_, ≥99.9%)	Sigma-Aldrich	Cat# 223891
Tellurium dioxide (TeO_2_, ≥97%)	Sigma-Aldrich	Cat# 86370
Nitric acid (HNO_3_, 65%)	Merck	Cat# 100456
Cabon black nanoparticles	Lion Specialty Chemicals	EC600
Poly(diallyldimethylammonium chloride) (PDDA, 20 wt %	Sigma-Aldrich	Cat# 409014

### Experimental model and study participant details

Omitted as our study does not involve biological models.

### Method details

CB/Bi_2_Te_3_ nanocomposite films were synthesized by a potentiostatic electrodeposition system with a three-electrode cell. The auxiliary and reference electrodes used in this study were Pt strip and silver/silver chloride (Ag/AgCl), respectively. A glass substrate with chromium-gold (Cr-Au) seed layer as a working electrode was immersed in the electrolyte vertically and the distance between the substrate and counter electrode was fixed at 4 cm. The substrate was prepared by sputtering the thin film of Cr-Au using a magnetron sputtering machine. The aqueous solution of the electrolyte was prepared by dissolving the bismuth oxide (Bi_2_O_3_) and tellurium dioxide (TeO_2_) powders in 1.0M nitric acid (HNO_3_). The mixing process was done in an ultrasonic bath to enhance the process of dissolving the powders. The electrolyte solution that consisted of 3.2 mM Bi^3+^ and 7.2 mM HTeO_2_^+^ was blended with desired concentration of porous CB-Nps at 0.20 to 0.33 g/L. Further blending process was applied for 1 h of stirring after the blended electrolyte being sonicated for approximately 2 h. During the stirring process, the electrolyte was continuously purged by N_2_ gas for removing dissolved oxygen.

The commercially made porous CB-Nps (>99% purity, Lion Speciality Chemicals, EC600) used in this study is usually hydrophobic and has a size of about 34 nm.^60^ The existance of the porous structure of the CB-Nps attributes to the low thermal conductivity. The CB-Nps was greatly difficult to suspend even temporarily in the aqueous solution. The suspension problem is related to the stability of the dispersed CB-Nps in the solution. The instability is caused by the agregation becouse of van der Waals attraction forces between particles.^61^ The particles will be attracted to each other when the attraction force dominates. Therefore, to avoid this attraction, the CB-Nps need to be graphted with Poly(diallyldimethylammonium chloride) (PDDA) as a comparable surround repulsion force to initiate stability. Firstly, the CB-Nps is mixed with a dissolved PDDA solution by stirring and sonicating intermittently for up to 1 h. During this process, the CB-Nps surface was attached by polymeric molecules of PDDA and became sterically stabilized. Additionally, further stability of dispersed particles has been achieved with free polymer molecules in the dispersion particles which is known as depletion stabilization.^62^^,^^63^ The excessive polymer was then reduced generously by filtration process. Finally, the filtered polymeric coated CB-Nps is blended with the prepared acidic electrolyte that contain the bismuth and telluride ions. Additional details of the observational study on the effect of CB-NP dispersion are provided in the Supplementary Information (Data S2).

Electrochemical co-depositions were potentiostaticly carried out at −90 mV in a pulse deposition method with no applied heating on the electrolyte solution. The targeted thickness of the deposited films was controlled at 3 to 4 μm. The pulse cycle consists of an applied potential, *E*_*on*_ at a period, *t*_*on*_ and an applied potential, *E*_*off*_ at a period, *t*_*off*_. A rapid co-deposition occurred during 100 ms of *t*_*on*_ at applied potential −90 mV, *E*_*on*_. No deposition happened during *E*_*off*_ period. The duty cycle (*t*_*on*_*/*(*t*_*on*_ *+ t*_*off*_)) was maintained at 33% for all deposition. To reduce the possibility of oxygen impurity incorporation in the nanocomposite film, the electrolyte was continuously blown by N2 gas throughout the electrochemical deposition process.

All synthesised films were annealed under N_2_ atmospheric pressure at 250°C for 2 h before they went through any properties measurement. The Seebeck coefficient and the electrical conductivity of samples were measured at room temperature by steady state method of a Seebeck measurement system and a collinear DC four-probe device (K-705RS Kyowariken), respectively. All the measurements were performed on the synthesized films that were transferred onto the molded epoxy as an insulator layer. The Seebeck measurement system was meticulously calibrated with the electrodeposited film of Bi_2_Te_3,_ as reported by Trung et al.^64^ Meanwhile, the carrier concentration was calculated from the Hall coefficient measured according to ASTM International F76-86.

The density of the films was determined using mass and volume measurements. The mass of each film was measured using an analytical balance with a resolution of 0.001 mg. Film thickness was obtained from cross-sectional measurements using scanning electron microscopy (SEM), and the average thickness was calculated from multiple positions across each sample. The film area was measured optically, and the density was calculated from the measured mass and geometric volume.

For thermal conductivity measurement, a test specimen is prepared by electrodepositing the nanocomposite film on a silicon substrate with chromium-gold (Cr-Au) seed layer. The substrate was prepared by sputter deposion of the thin Cr-Au films on 200 μm-thick silicon substrate. Thermal conductivity of deposited films was measured by a differential 3-omega (3ω) method.^65^^,^^66^^,^^67^ The differential 3ω method requires two specimens; one is sample specimen with the deposited film and the other is the reference specimen without the film. Fundamentally, the 3ω method utilizes the signal of third harmonic voltages (3ω-voltages) generated at a microfabricated Cr-Au heater pattern on the film surface. The specimens were prepared by depositing a 600 nm thin SiO_2_ film and a Cr (20 nm)-Au (300 nm) films on top of the specimen’s surface. The pattern of the heater with a length 1.0 mm and width 50 μm was formed by photolithography and wet etching processes. The heater also simultaneously operated as a thermometer to measure the temperature variation. Details of required equations and measurement procedure were followed as Zhou et al.^68^ Details of the measurement procedures and error mitigation are provided in the supplementary information (Data S3).

The hardness masurement has been performed by using an ultramicro-indentation device. The indentation system utilizes a continuous stiffness measurement (CSM) method on the micro-thick film where the continuous measuring load and displacement are recorded during the constant indentation process up to a specified maximum load. The constant indentation speed can be applied as low as 5 nm/s with a Berkovich diamond indentor. Meanwhile the Young’s modulus value was estimated through the measured stiffness of the unloading data from the hardness measurement.^57^^,^^69^^,^^70^ The surface and cross-sectional morphologies were observed by scanning electron microscopy (SEM) for crystallite formation investigation and thickness determination, respectively. Meanwhile, the element composition of the deposited film was analyzed using energy dispersive X-ray spectroscopy (EDX). Further study on the elements composition and the crystal size were carried out using X-ray diffraction (XRD) analysis. The average grain size was calculated through the calculation of Scherer equation. The nanocomposite films were analyzed by high resolution TEM (HRTEM), selected area electron diffraction (SAED) and weak-beam dark field (WBDF).

### Quantification and statistical analysis

Omitted-There are no quantification or statistical analyses to include in this study.

No formal statistical analyses were performed in this study. Graphs were generated using Microsoft Excel based on the mean values obtained from repeated measurements. The central tendency is represented by the arithmetic mean, while variability is represented by the measurement range (minimum–maximum values) shown as error bars. No hypothesis testing, significance testing, randomization, stratification, sample size estimation, or exclusion criteria were applied.

### Additional resources

Omitted-There are no additional resources.

## Footnotes

Supplemental information can be found online at https://doi.org/10.1016/j.isci.2026.116977.
