# Supplementary material for: Electrodeposited Bi2Te3-carbon black nanocomposites with enhanced thermoelectric and mechanical properties
Source: iScience. 2026 Aug 22;29(9):116977. doi: 10.1016/j.isci.2026.116977 (PMC13524735; doi:10.1016/j.isci.2026.116977)
Supplement: Supplementary file 1 [file mmc1.pdf]

iScience, Volume *29*

Supplemental information

Electrodeposited Bi_2Te_3-carbon black nanocomposites with enhanced thermoelectric and mechanical properties

Khairul Fadzli Samat, Muhammad Afiff Alias, Muhammad Aliff Ikhwan Che Azman, Mohd Hafiz Jali, Nguyen Van Toan, and Takahito Ono

Data S1. Figure S1

DATA S1: ELEMENTAL MAPPING ANALYSIS

Elemental mapping of CB/Bi_2Te_3 film

Figure S1 presents EDX elemental mapping of the CB/Bi_2Te_3 films, demonstrating a uniform distribution of Bi, Te, and carbon across the film surface. The carbon map confirms that CB-NPs are well dispersed within the Bi_2Te_3 matrix, with no evident large-scale agglomeration. This homogeneous CB-Nps incorporation supports the formation of distributed nanoscale scattering centers, which are beneficial for suppressing phonon transport through enhanced interface and defect scattering.

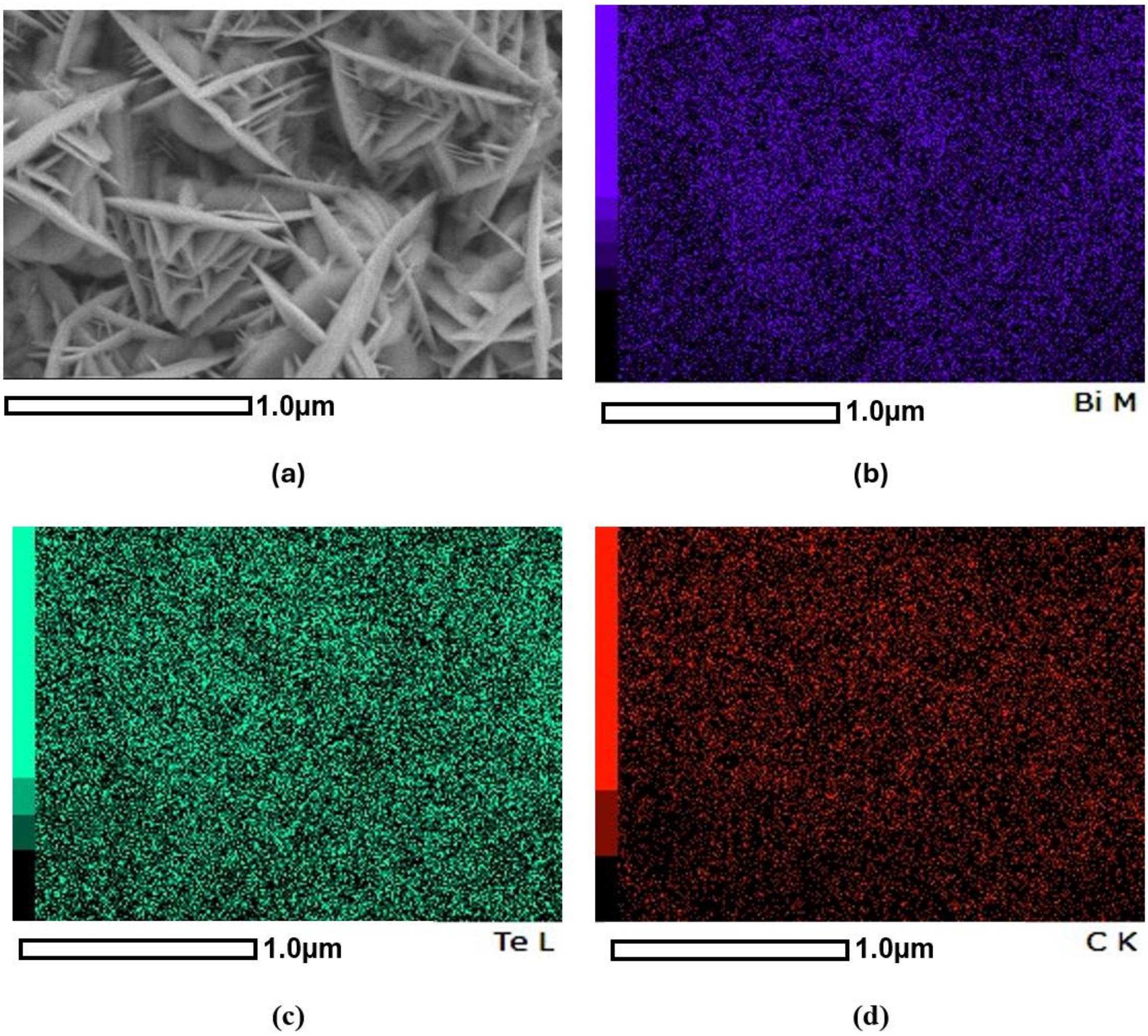

Figure S1: Elemental mapping of the CB/Bi_2Te_3 film: (a) SEM image of the film surface, and corresponding EDX maps of (b) bismuth (Bi), (c) tellurium (Te), and (d) carbon from carbon black nanoparticles (CB-NPs). The scale bar shown in image corresponds to 1.0µm.

Data S2. Figures S2-S3, Table S1 and supplemental references

DATA S2: OBSERVATIONAL STUDY OF THE EFFECT OF CB-NP DISPERSION

Improvement of CB-NP Dispersion and Suspension in the Electrolyte

Figure S2 illustrates the improvement in dispersion and suspension stability of carbon black nanoparticles (CB-NPs) in the electrolyte solution after CB-NPs polymeric modification. The modified electrolyte exhibits enhanced colloidal stability, primarily due to the reduced influence of van der Waals attractive forces between CB-NPs [1]. This reduction is achieved through polymer adsorption on the CB-NPs surfaces, where the coated polymer layer provides steric repulsion that counterbalances interparticle attraction. In addition, free polymer chains present between neighboring nanoparticles induce depletion stabilization, further suppressing aggregation and sustaining a homogeneous dispersion [2].

The dispersion stability of CB-NPs in the electrolyte was systematically evaluated under static conditions, with observations recorded at 30-min intervals and beyond 1 hour without mechanical stirring or sonication (Table S1). For the electrolyte without modified CB-NPs, rapid aggregation and clear sedimentation were observed within 30 min, indicating poor dispersion stability. In contrast, the electrolyte containing modified CB-NPs exhibited a well-dispersed and homogeneous suspension with no noticeable sedimentation up to 90 min and beyond. These observations confirm that the improved electrolyte effectively enhances CB-NP dispersion and suspension stability under static conditions, which is critical for achieving uniform nanoparticle incorporation during subsequent electrochemical co-deposition processes.

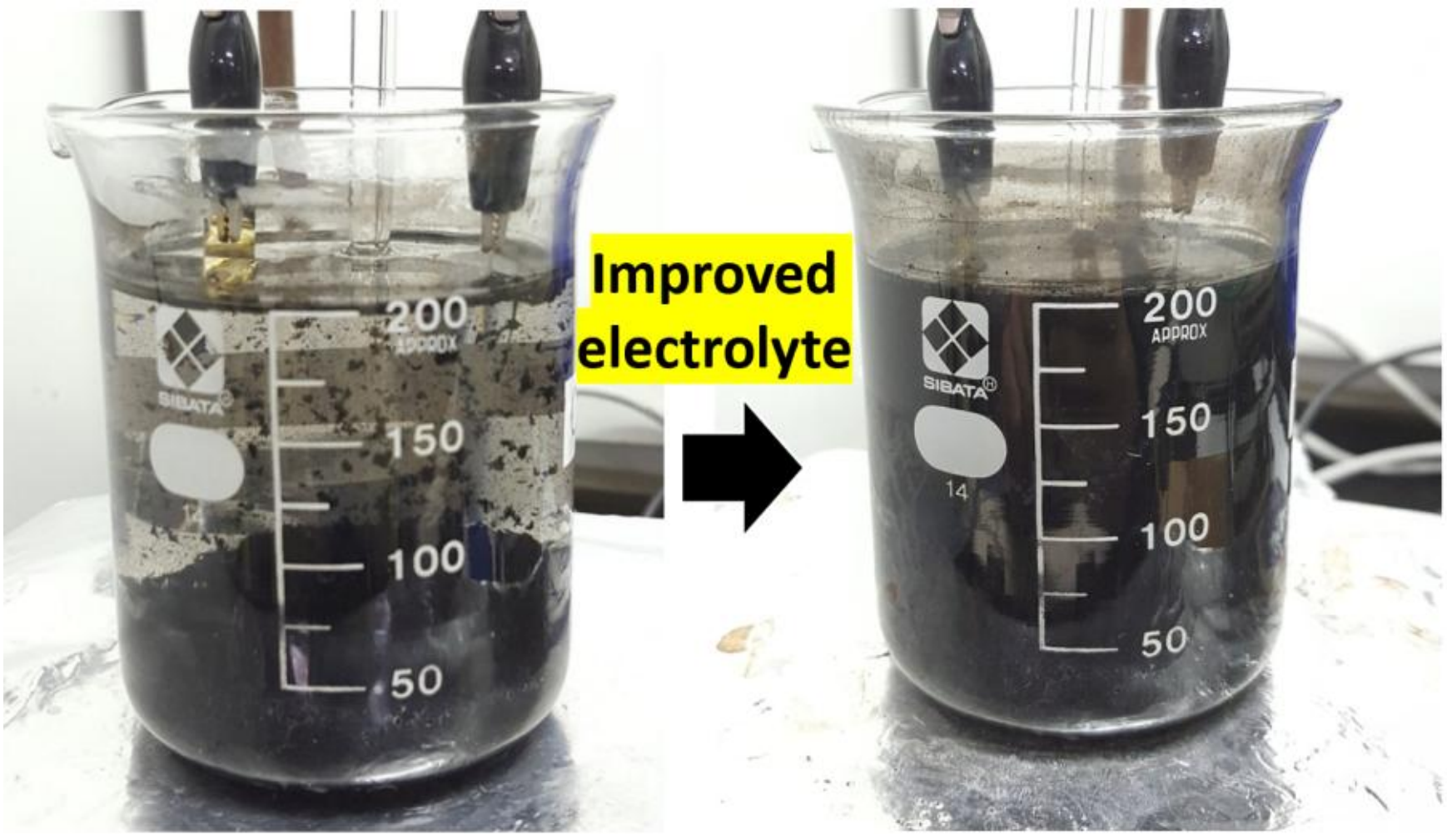

Figure S2: Improved electrolyte condition after enhancement of CB-NP dispersion and suspension stability.

Table S1: Observation of CB-NP dispersion and suspension stability in the electrolyte under static conditions. No mechanical stirring or sonication was applied during the observation period.

Electrolyte condition	Time of Observation	Dispersion and suspension observation
Electrolyte without modified CB-NPs	30 min	Poorly dispersed and clear sedimentation of CB-NPs
Electrolyte with modified CB-NPs	30 min	Well dispersed and no noticeable sedimentation.
	60 min	
	90 min	
	>90 min	

The effect CB-Nps deposition on film

The influence of CB-NP dispersion quality on the deposited CB/Bi_2Te_3 nanocomposite film is shown in Figure S3. Films deposited using the improved electrolyte exhibit a more uniform distribution of CB-NPs with significantly reduced nanoparticle aggregation. In contrast, deposition from a less stable electrolyte results in pronounced CB-NP agglomeration within the film, leading to inhomogeneous microstructures. At higher magnification, CB-NPs encapsulated within the Bi_2Te_3 matrix appear to be well dispersed, showing substantially reduced aggregation behavior. The improved dispersion therefore plays a critical role in achieving uniform CB incorporation during electrodeposition, which is essential for consistent electrical and thermal transport properties.

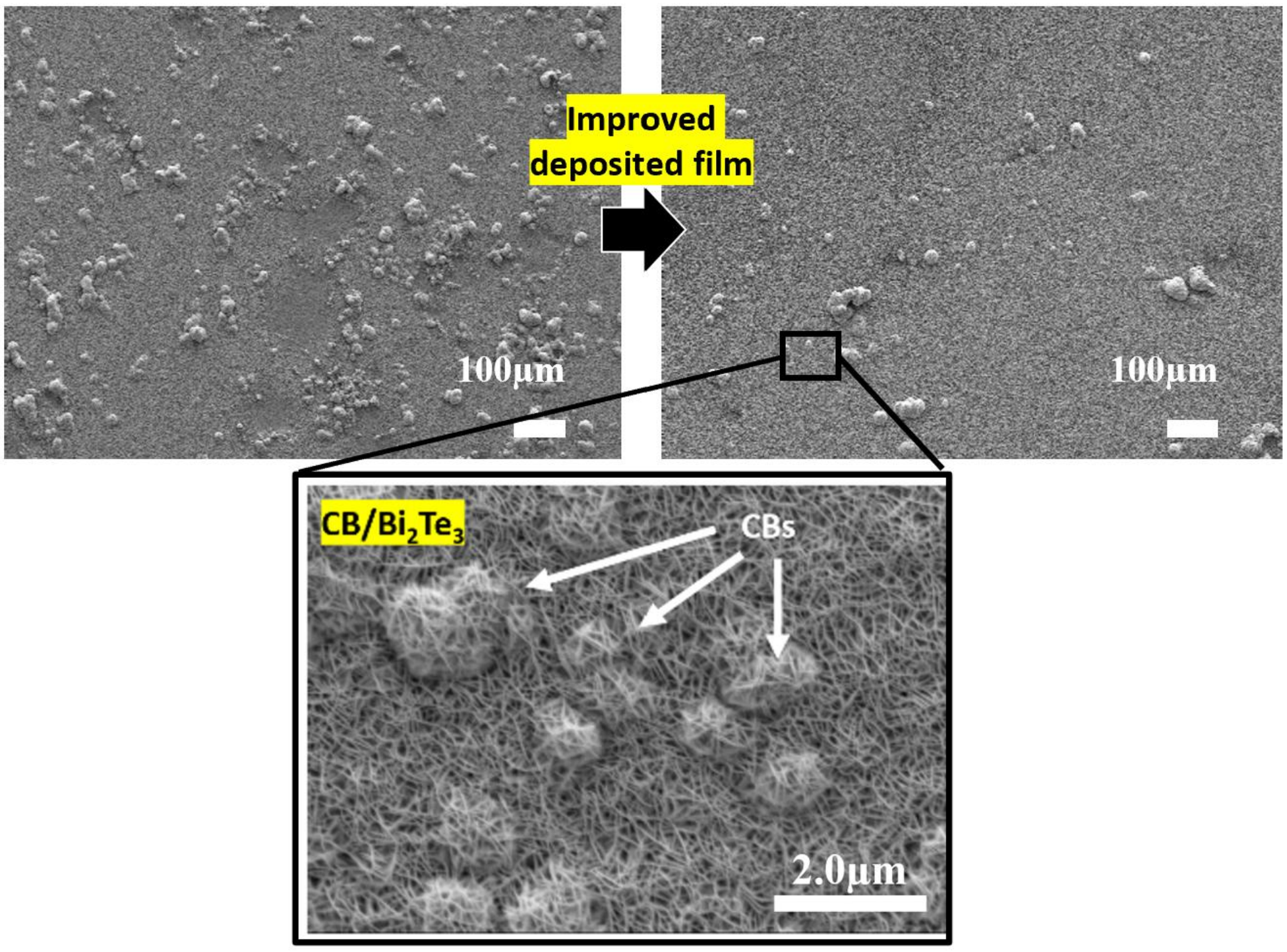

Figure S3: Comparison of CB/Bi_2Te_3 films deposited using (left) poorly dispersed and (right) improved CB-NP electrolyte conditions. Enhanced CB dispersion leads to reduced aggregation and more uniform nanoparticle distribution.

Supplemental references

[1] Tadros, T. (2012). Electrostatic and steric stabilization of colloidal dispersions. In *Electrical Phenomena at Interfaces and Biointerfaces*, 153–172. https://doi.org/10.1002/9781118135440.ch10

[2] Min, Y., Akbulut, M., Kristiansen, K., Golan, Y., and Israelachvili, J. (2008). The role of interparticle and external forces in nanoparticle assembly. Nat. Mater. 7, 527–538. https://doi.org/10.1038/nmat2206

Data S3. Figure S4, Table S2

DATA S3: THERMAL CONDUCTIVITY MEASUREMENT

Measurement Principle

The thermal conductivity of the deposited thin films was measured using a differential 3-omega (3ω) method, which is well established for thin-film thermal characterization due to its high sensitivity and capability to reduce systematic errors associated with substrate contributions. All measurements were conducted under vacuum conditions at room temperature to suppress convective heat losses and ensure thermal stability.

In the 3ω technique, a micro-fabricated metal heater serves simultaneously as a Joule heat source and a resistance thermometer. When an alternating current at angular frequency ω is applied to the heater, resistive heating occurs at 2ω, inducing a periodic temperature oscillation at the same frequency. This temperature fluctuation causes a resistance modulation that generates a voltage component at the third harmonic (3ω), which is directly related to the temperature rise of the heater.

To isolate the thermal response of the deposited film from that of the substrate, a differential configuration was employed. Two nominally identical specimens were prepared:
(i) a film specimen, consisting of the substrate with the deposited film, and
(ii) a reference specimen, consisting of the same substrate structure without the deposited film.
By comparing the temperature responses of these two specimens, the thermal contribution of the film alone can be reliably extracted.

Specimen Preparation and Micro-Heater Fabrication

The thermal conductivity of the deposited thin films was measured using a differential 3-omega (3ω) method, which is well established for thin-film thermal characterization due to its high sensitivity and capability to reduce systematic errors associated with substrate contributions. All measurements were conducted under vacuum conditions at room temperature to suppress convective heat losses and ensure thermal stability. Both film and reference specimens were fabricated following identical processing steps to minimize systematic discrepancies. A dielectric SiO_2 layer was first deposited onto the specimen surface, followed by a Cr/Au metal layer to form the micro-heater. The SiO_2 layer electrically isolated the heater from the underlying material and ensured stable operation.

The micro-heater pattern was defined using standard photolithography and wet-etching processes. The heater geometry was designed with a length of 1.0 mm and a width of 50 μm, ensuring one-dimensional heat flow along the film thickness during measurement. Electrical connections were established using 50-μm-diameter Au wires bonded with silver paste, and the specimen was mounted on a copper plate to provide mechanical stability and effective heat sinking.

A summary of the micro-fabrication process parameters is provided in Table S2.

Table S2: Summary of the micro heater fabrication process

**Process**	**Related Methods and Required Parameter**
1. Thermoelectric material deposition	Electrodeposition process<br>(Thickness: 1-2μm)
2. SiO_2 deposition	Plasma-enhanced TEOS CVD (P-TEOS CVD)<br>(Thickness: 300 nm)
3. Cr-Au deposition	RF Magnetron Sputter (Shibaura#2)<br>(Thickness; Cr: 20nm, Au: 200nm)
4. Photolithography	Spin-coating of OFPR-200cp<br>(Thickness: ~5 μm)
5. Cr-Au etching & photoresist cleaning	• Wet etching by Cr/Au etchants<br>• Photoresist cleaning (Acetone + Ethacol)

Electrical Measurement Configuration

The experimental setup consisted of a function generator, a lock-in amplifier, an oscilloscope, and an integrated Wheatstone bridge circuit. The Wheatstone bridge was employed to suppress the dominant first-harmonic (1ω) voltage and enhance the detection sensitivity for the much smaller third-harmonic (3ω) signal, which is typically three orders of magnitude lower than the fundamental voltage. Under the balanced Wheatstone bridge conditions, minimal current flows through the bridge arm connected to the lock-in amplifier, effectively reducing background noise.

To ensure that nearly all the supplied current passed through the micro-heater, the resistance $R_2$was chosen to be at least 2000 times larger than R_h, limiting current leakage through the bridge to below 0.2%. As a result, more than 99.8% of the applied current flowed through the heater branch, allowing reliable extraction of the heater's harmonic voltages. The first-harmonic heater voltage is given by,

$$V_{h,1/2\omega} = \frac{R_h}{R_h + R_{1/2}} V_s,$$

where V_s is the source voltage supplied by the function generator, $R_h$is the heater resistance, $R_{1/2}$ is fixed resistor.

Temperature Extraction and Differential Analysis

The micro-heater resistance varies linearly with temperature within the small oscillation range employed in this study. The temperature rise of each specimen was obtained from the ratio of the third-harmonic to first-harmonic voltages:

$$\Delta T_{1,2} = \frac{2R_0}{dR/dT}\left(\frac{V_{h,3\omega}}{V_{h,1\omega}}\right),$$

where $R_0$is the heater resistance at room temperature and $dR/dT$is the temperature coefficient of resistance (TCR) of the Cr/Au heater. The effective temperature rise, ΔT_f associated solely with the deposited film was determined using differential temperature analysis as follows,

$$\Delta T_f = \Delta T_1 - \left(\frac{P_1}{P_2}\right)\Delta T_2,$$

where $\Delta T_1$and ΔT_2correspond to the temperature rises of the film and reference specimens, respectively, and $P_1$and P_2denote the electrical power dissipated in the heater for each specimen. Figure S4 shows a representative example of the effective temperature difference as a function of measurement frequency for films with different CB contents.

This differential approach significantly mitigates uncertainties related to substrate thermal properties, heater geometry, and parasitic heat losses.

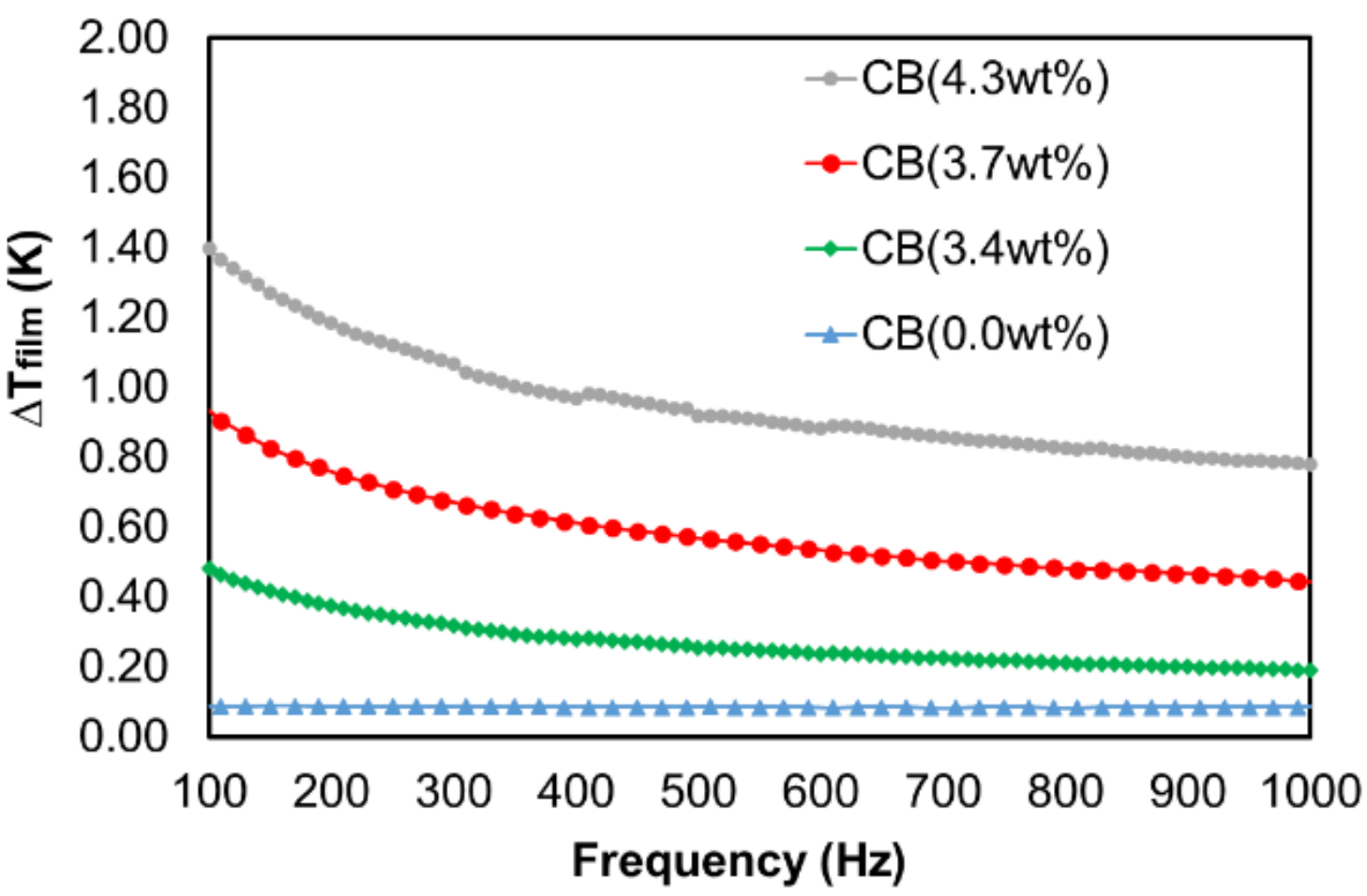

Figure S4: An example of the calculated temperature different of film as a function of frequency

Thermal Conductivity Calculation

The cross-plane thermal conductivity of the deposited film was calculated as,

$$\kappa = \frac{P_1 t_f}{L w \Delta T_f},$$

where t_f is the film thickness, and L and w are the length and width of the micro-heater, respectively. Film thickness was independently measured by cross-sectional scanning electron microscopy (SEM), and multiple measurements were averaged to minimize thickness-related uncertainty.

Measurement Accuracy and Error Mitigation

Several measures were taken to ensure the reliability and reproducibility of the thermal conductivity data:

1. Identical heater geometries and fabrication processes were employed for all specimens to ensure consistency.
2. Vacuum measurement conditions minimized convective heat losses.
3. Differential specimen design reduced substrate-related systematic errors.
4. Wheatstone bridge balancing suppressed large background signals and enhanced sensitivity to nanovolt-level 3ω signals.
5. Lock-in detection allowed selective measurement of the 3ω component with high signal-to-noise ratio.

Repeated measurements confirmed good reproducibility, and no abnormal frequency dependence or signal instability was observed within the measurement range.
